# Weight Status Change in Chinese American Children over a Ten-Year Period: Retrospective Study of a Primary Care Pediatric Population

**DOI:** 10.3390/ijerph19105916

**Published:** 2022-05-13

**Authors:** Jia Lu Lilian Lin, Olivia Zhong, Raymond Tse, Jennifer D. Lau, Eda Chao, Loretta Au

**Affiliations:** 1 Institute of Health Policy, Management and Evaluation, University of Toronto, Toronto, ON M5T 3M6, Canada; jialu.lin@mail.utoronto.ca; 2 CUNY School of Medicine, New York, NY 10031, USA; ozhong000@citymail.cuny.edu; 3 Charles B. Wang Community Health Center, New York, NY 10013, USA; rayt331@gmail.com (R.T.); jennifer.lau09@gmail.com (J.D.L.); echao@cbwchc.org (E.C.); 4 Weill Cornell Medicine, New York, NY 10065, USA

**Keywords:** Asian Americans, overweight, obesity, pediatrics, emigrants and immigrants

## Abstract

Weight change from childhood to adolescence has been understudied in Asian Americans. Known studies lack disaggregation by Asian subgroups. This retrospective study assessed the weight status change in 1500 Chinese American children aged 5–11 years from an urban primary care health center between 2007 and 2017. Weight status was categorized using the 2000 CDC growth charts into “underweight/normal weight” and “overweight/obese.” The overweight/obesity prevalence in 2007 and 2017 were determined. McNemar’s test and logistic regression were performed. The prevalence of overweight/obesity decreased from 29.9% in 2007 to 18.6% in 2017. Children who were overweight/obese at 5–11 years had 10.3 increased odds of staying overweight/obese over time (95% CI = 7.6–14.0, *p* < 0.001) compared to their underweight/normal weight counterparts. Of the children who were overweight/obese in 2007, 45.7% remained overweight/obese ten years later. Childhood overweight/obesity strongly predicts adult overweight/obesity in Chinese Americans. Targeted education and intervention are warranted to prevent adult obesity.

## 1. Introduction

Childhood obesity continues to be a major public health crisis in the United States, where approximately 19.3% of children and youth aged 2–19 years are obese, and another 16.1% are overweight, according to national estimates from 2017–2018 [1,2]. Childhood and adolescent obesity increase the risk of long-term comorbidities, including type 2 diabetes, stroke, coronary heart disease, hypertension, and premature mortality [3,4].

Several studies show that overweight and obesity in children are important predictors of adult overweight or obesity [5,6,7,8]. According to a 2016 systematic review and meta-analysis of 15 large prospective cohort studies, obese children and adolescents were around five times more likely to be obese as adults than children/adolescents who were non-obese [9]. Analyses of racial/ethnic differences in the maintenance of childhood body mass index (BMI) into adulthood found that Black overweight children had a greater predictive value for adult obesity than their White counterparts [10]. While some studies have highlighted racial/ethnic differences in obesity prevalence with increasing age [10,11,12], they have not examined differences in changes in weight status from childhood to adulthood between Asian Americans and other racial/ethnic groups. 

Compared to all other races and ethnicities, Asian American children and adolescents aged 2–19 years have significantly lower prevalence of overweight (23.2%) and obesity (10.7%) [12]. However, Asian adult populations have a higher body fat percentage at a lower BMI compared to Whites [13], and greater disease and mortality risks than other racial/ethnic groups at the same BMI [14,15]. A few studies have suggested that using the standard BMI cutoff points to define overweight/obesity (BMI ≥ 25 kg/m^2^) may be inappropriate for Asian Americans, since their obesity-related health risks may be underestimated [16,17,18]. Moreover, while obesity prevalence is higher among adolescents (ages 12–19) compared to children (ages 6–11) in the White, Black, and Hispanic populations, this trend is reversed among Asian Americans [19,20,21]. The knowledge gaps regarding obesity trends and obesity-related health risks in Asian Americans reveal an urgent need to understand the trends in overweight and obesity among Asian Americans compared to other racial/ethnic groups. This study examined weight status change from childhood to adolescence/young adulthood in a cohort of Chinese American children aged 5–11 years from one primary care health center.

Two cross-sectional studies, conducted in 2004 and 2015, showed a decrease in the overweight/obesity prevalence, from 24.6% to 21%, among Chinese American children who received care at a New York City (NYC) community health center that serves a large population of low-income Chinese Americans [22,23]. Although these cross-sectional studies both employed electronic health record (EHR) data from the same clinical population, it was unknown whether a decrease in overweight/obesity prevalence would similarly be found in a single cohort of children followed during this time period. In the present study, we conducted a retrospective review of a cohort of Chinese American children from the same community health center between 2007 and 2017. This study aims to: (1) examine the change in weight status categories over ten years in a cohort of Chinese American children receiving care at one health center; and (2) determine whether overweight/obesity in childhood predicts adult overweight/obesity among Chinese Americans.

## 2. Materials and Methods

### 2.1. Study Design and Participants

We conducted an EHR-based retrospective cohort study of 1500 ethnically Chinese school-aged children (5–11 years old) who had their BMI percentiles assessed at annual preventive health visits in both 2007 and 2017 at the Charles B. Wang Community Health Center (CBWCHC), a Federally Qualified Health Center serving a predominantly Chinese immigrant population in NYC since 1971. The CBWCHC has a sliding fee scale to assist self-paying patients without access to health insurance as well as accepts publicly sponsored health insurance eligible for low-income families meeting poverty level guidelines. In 2017, the NYC poverty threshold was USD 33,562 for two-adult, two-child families (19.0 percent poverty rate) [24]. Meanwhile, in comparison, NYC’s non-Hispanic Asian poverty rate was 23.8 percent [24], and its noncitizen poverty rate was 26.7 percent [25].

Children were eligible for study inclusion if the following criteria were met: (1) 5 to 11 years of age in 2007; (2) ethnically Chinese (i.e., people of Chinese descent); (3) had a primary care pediatrician at the CBWCHC; and (4) had an annual preventive health visit at the CBWCHC in both calendar years 2007 and 2017.

**Figure 1:**
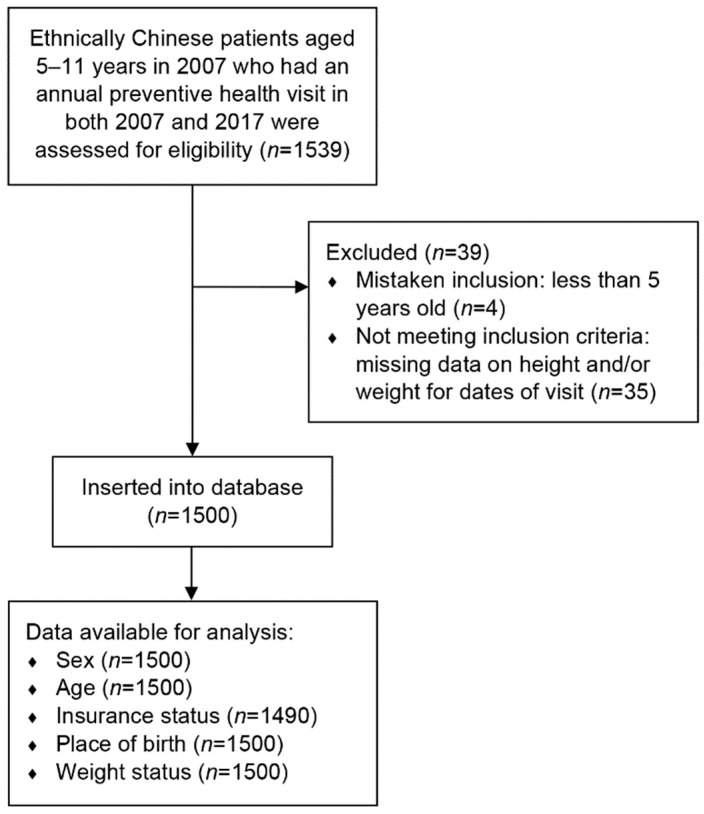
Study flow.

Children were excluded if their height or weight data were missing for the dates of visit. Figure 1 shows a flowchart of the present study.

### 2.2. Ethical Considerations

This study was reviewed by the Sterling Institutional Review Board (IRB) and was determined to be exempt. Since there is no local IRB at the CBWCHC, ethical approval was sought from Sterling IRB, an independently owned and operated IRB whose clients include federal agencies and individual investigators, pharmaceutical and biotechnology companies, and institutions such as medical centers, academic institutions, and community hospitals [26].

### 2.3. Measures

Overweight and obesity status were determined using the BMI percentiles based on the 2000 Centers for Disease Control and Prevention (CDC) growth charts [27]. In children and adolescents, CDC defines overweight as having a BMI equal to or greater than 85th percentile and less than 95th percentile, and obese as equal to or greater than 95th percentile in relation to other children and adolescents of the same age and sex [27]. Among those who are 20 or 21 years old, overweight is defined as having a BMI equal to or greater than 25 kg/m^2^ and less than 30 kg/m^2^, and obese as having a BMI equal to or greater than 30 kg/m^2^ [27].

At each preventive health visit, clinically trained nursing staff measured and recorded the child’s weight and height into the patient’s EHR record (Athenahealth EHR Software, Watertown, MA, USA). BMI was computed using the formula: weight (kg)/height^2^ (m^2^). Exact BMI percentiles were calculated by the CDC growth chart formula embedded in EHR.

### 2.4. Statistical Analysis

Statistical analyses were performed using IBM SPSS Statistics version 25 [28]. Participants were stratified into two groups for weight status: underweight/normal weight (BMI percentile < 85% if less than 20 years of age, or BMI < 25 kg/m^2^ if 20 years or older), or overweight/obese (BMI percentile ≥ 85% if less than 20 years of age, or BMI ≥ 25 kg/m^2^ if 20 years or older). Participants were also categorized into two groups for place of birth (US-born, foreign-born), two groups for age (5–8, 9–11 years), and four groups for insurance status (public insurance or self-pay at both time points, private insurance at both time points, switched from public to private insurance, switched from private to public insurance).

Frequency distributions of categorical data were calculated. Mean and standard deviation were described for continuous data. The overall prevalence of overweight/obesity in 2007 and 2017 were determined. Prevalence rates were calculated for boys and girls, age groups 5–8 and 9–11, US-born and foreign-born, public insurance and private insurance, independently for 2007 and 2017. Since paired nominal data were used, the McNemar’s test was performed to assess whether the proportion of overweight/obesity in 2007 is different from that in 2017. A full logistic regression model was estimated including age group, sex, insurance status, and place of birth to test for confounders and interaction effects. The final logistic regression models, stratified by the interaction term and adjusted for confounders, were used to estimate associations between overweight/obesity at the two time points. The positive predictive value was calculated as the percentage of those overweight/obese in 2007 who remained overweight/obese in 2017. Furthermore, we calculated the percentage of those underweight/normal weight in 2007 who became overweight/obese in 2017. A *p*-value below 0.05 was considered statistically significant.

## 3. Results

In 2007, a total of 1500 children ages 5–11 years included in the study were 51% male and 49% female (Table 1). A greater proportion (73.9%) of the participants were aged 5–8 years, with 26.1% aged 9–11 years in 2007. Among the four groups categorized by insurance status, most participants (82.7%) were grouped in the public insurance/self-pay category at both the beginning and end of the study period.

The overall prevalence of overweight and obesity in the study sample dropped from 29.9% in 2007 to 18.6% in 2017 (Table 2). In both 2007 and 2017, the overweight/obesity prevalence in boys (from 38.4% to 24.3%) was twice that of girls (from 21.1% to 12.6%). While declines in overweight/obesity prevalence were seen in both the US-born and foreign-born subgroups, the US-born subgroup had a slightly higher overweight/obesity prevalence in both 2007 and 2017, and a steeper decline in overweight/obesity prevalence compared to the foreign-born subgroup. For the overall study sample and every sample characteristic (e.g., age, place of birth, insurance status), the change in proportion of overweight/obese status from 2007 to 2017 was statistically significant, *p* < 0.001 (Table 3).

From 2007 to 2017, nearly half (45.7%) of children who were overweight/obese at the start of the study remained overweight/obese in 2017. However, only a minority (7.0%) of those who were normal weight/underweight in 2007 became overweight/obese in 2017. As shown in Table 4, those who were overweight/obese in 2007 had a much greater likelihood of remaining overweight/obese in 2017 compared to the likelihood of those who were normal weight/underweight in 2007 to become overweight/obese in 2017. Sex was found to be associated with weight status and was adjusted for in all the models, *p* < 0.001 at both time points. In our sample, children who were overweight/obese in 2007 had 10.3 times more odds of staying overweight/obese in 2017 after adjusting for sex (95% CI = 7.6–14.0, *p* < 0.001). Interaction between staying overweight/obese and place of birth was observed, *p* < 0.01. Separate logistic regression models were estimated for US-born and foreign-born children. Foreign-born overweight/obese children had 40.6 times more odds (95% CI = 14.3–115.4, *p* < 0.001) of staying overweight/obese in adolescence/young adulthood compared to foreign-born underweight/normal weight children. In contrast, US-born overweight/obese children had 8.6 times the odds (95% CI = 6.3–11.9, *p* < 0.001) of staying overweight/obese in adolescence/young adulthood compared to US-born underweight/normal weight children.

## 4. Discussion

In this retrospective cohort study of 1500 Chinese American children, overweight/obesity prevalence over a ten-year span dropped from 29.9% (ages 5–11) to 18.6% (ages 15–21). This finding departed from the common trend of increasing overweight/obesity prevalence from childhood to adolescence/young adulthood reported in studies conducted in the United States [10,29,30] and Northern Norway [8]. In comparison, Cunningham and colleagues’ study using data from a large, nationally representative cohort of children in the US who were followed from ages 5 to 14 years reported increases in overweight/obesity prevalence over nine years for all racial/ethnic groups: an increase from 23.9% to 32.4% in non-Hispanic White children; increase from 29.7% to 45.8% in non-Hispanic Black children; increase from 35% to 47.5% in Hispanic children; and increase from 29.3% to 37.1% in children of other races (Asian, Pacific Islander, Native American, and multiracial children) [29]. The discrepancies between the results of our study and that of Cunningham et al.’s study may be attributable to the fact that Chinese American children had the lowest prevalence of overweight (11.8%) among Asian ethnic groups, based on analyses of the 2011–2014 National Health and Nutrition Examination Survey (NHANES) data [21].

However, there have also been studies that found a decrease in overweight/obesity prevalence as children and adolescents grew older [31,32]. Our findings are consistent with that of Araújo et al.’s study of Portuguese adolescents [31], which reported more adolescents switching to a lower weight category over time than those who switched to a higher weight category. However, Araújo et al.’s study had only a 38-month follow-up period [31]. In the current study, the combination of a long (120-month) study period and a large sample of children (*n* = 1500) who received care from the same health center afforded control over social environmental factors and assured that all study participants had similar exposure to health messages and targeted interventions. Therefore, we can more confidently attribute our primary findings to an overall pattern of improved weight control over the ten-year study period.

Despite the overall decrease in overweight/obesity prevalence in this Chinese American pediatric population, our findings confirm existing knowledge on the persistence of overweight/obesity from childhood to adulthood [6,10,33,34]. In our study, children who were overweight/obese at ages 5–11 had 10.3 times more odds of becoming overweight/obese at ages 15–21, compared to children who were underweight/normal weight. Odds ratios similar to our study were reported by studies on overweight/obesity tracking from childhood to adolescence conducted in Norway [8] and Iceland [7]. The current study found that 45.7% of overweight/obese children at baseline (48.5% of boys and 40.3% of girls) were still overweight/obese ten years later. The percentage of overweight/obese children who remained overweight/obese as adolescents/young adults in our study is lower compared to studies conducted in Louisiana (68%) [10], Norway (63%) [8], and Iceland (51%) [7], but was significantly higher than that found in a Chinese study (6.7%) [35]. When stratified by sex, our study’s positive predictive value is lower compared to a Slovenian study (over 65% among boys and below 50% among girls) [6], but on par with a Portuguese study (51.5% among boys and 41.9% among girls) [31]. These variations in positive predictive values may be due to differences in the ages at baseline and follow-up, duration of follow-up, how overweight/obesity was defined, and environmental factors such as access to healthy foods, exposure to media and technology, and sedentary lifestyles. Our findings reaffirm the importance of implementing strategies for preventing obesity in early childhood [36,37].

As there is no globally standardized classification for overweight/obesity, it is plausible that variations in weight status change from childhood into adolescence/young adulthood reported in different studies are linked to the use of different BMI classification systems [38]. This study used the CDC growth charts with age- and sex-specific BMI percentile reference values for categorizing weight status. In contrast, similar studies conducted in Norway [8], Iceland [7], Slovenia [6], China [35], and Japan [39] used the International Obesity Task Force (IOTF) classification, while a Portuguese study [31] used the World Health Organization (WHO) classification. The IOTF reference provides age- and sex-specific BMI cutoff values for overweight and obesity in childhood that correspond with adult cutoff values of 25 kg/m^2^ and 30 kg/m^2^ for overweight and obesity, respectively [40]. The WHO child growth standards are based on an international sample of healthy breastfed infants and young children [41]. If our study had applied the WHO expert consultation report recommended BMI cutoffs for Asian adults [42] (at 23 kg/m^2^ for overweight and 27.5 kg/m^2^ for obesity) for participants aged 20 years or older, we may have observed a higher prevalence of overweight/obesity at the end of the study period [18].

This study found a higher likelihood of foreign-born children who were overweight/obese to stay overweight/obese in adolescence/young adulthood compared to those who were US-born. The literature on obesity risk factors among immigrant children in the US suggests that parental acculturation plays an important role in children’s obesity risk [43]. Less acculturated parents are more likely to work longer hours, have less time to prepare home-cooked meals and be less aware of obesity prevention and related resources, which may contribute to increased obesity risk for their children [43,44,45]. Since parental characteristics, such as parents’ place of birth, were not included in our analysis, it is inconclusive whether the observed increased likelihood of staying overweight/obese among foreign-born children could be explained by acculturation difference between the parents of US-born and foreign-born children in this study. Verifying this hypothesis requires further research, as the current literature shows mixed findings. One study of pre-school children living in low-income households in Los Angeles County reported that children with Chinese-speaking mothers who lived in Chinese immigrant enclaves had lower BMIs, suggesting a protective effect of Asian culture against childhood obesity [46]. Another study that used a nationally representative sample of US children did not find any significant relationship between acculturation levels and obesity risk except among non-Hispanic Black children [47]. More research is needed to understand how factors which may account for ethnic-nativity differentials in obesity risk, such as health care utilization, parental obesity and activity levels, dietary patterns, social environmental factors, and cultural differences, contribute to differences in obesity risk between US-born and foreign-born children [47].

The drop in overweight/obesity prevalence from 29.9% in 2007 to 18.6% in 2017 in Chinese American children in this study may be attributable to concerted efforts from local, state, and federal levels targeting childhood obesity [48,49,50] as well as CBWCHC’s comprehensive approach to managing childhood obesity. In particular, CBWCHC has since 2007 implemented an obesity registry to track children who are overweight or obese, and a patient-centered care management approach to offer culturally appropriate educational and nutritional counseling for pediatric patients who are overweight or obese [51,52]. The health center utilizes the primary care collaborative care model [53,54], which integrates multi-level strategies for managing chronic conditions, including evidence-based guidelines [55], team-based care, and patient and community engagement supported by organizational alignment and data analytics [56]. We speculate that while these strategies may have been effective in curbing overweight and obesity among the US-born in this study, they may have been less effective in reaching the foreign-born. We believe that the foreign-born children and adolescents may have been more focused on learning English and adapting to life in the US and were less willing to participate in community-based programs for nutrition and physical activity [43].

### Limitations

This study has several limitations. First, the sample was recruited from a single health center working with families from a lower socioeconomic status. Thus, on this ground, the results are not representative of Chinese American young people in the US and therefore not generalizable to all Chinese American communities. Second, since neither body fat percentage nor adiposity was measured in conjunction with BMI, which would have informed excess body weight in relation to height, overweight/obesity may have been underestimated using BMI alone [57]. Third, we did not collect data on diet, lifestyle, parental/family characteristics, participation in obesity interventions, or the age at which foreign-born children moved to the US, which would have better explained children’s change in weight status over time, particularly the differences seen among foreign-born versus US-born children. Fourth, we did not examine how each participant’s BMI percentiles had changed from 2007 to 2017 due to methodological difficulties involved in calculating adult BMI percentiles for participants aged 20 or 21 years. Despite these limitations, this study followed a large cohort of Chinese American children for ten years and provides a rare insight into changes in weight status categories from childhood to adolescence/young adulthood in this understudied ethnic population.

## 5. Conclusions

This study of weight status change in a large cohort of Chinese American children in NYC over ten years addresses a critical gap in the literature, namely understanding the patterns of entry into and exit from overweight and obesity amongst Chinese American children and adolescents. We demonstrated that childhood overweight/obesity is a strong predictor of overweight/obesity later in life. Targeted prevention and intervention efforts are warranted for the Chinese American pediatric population to prevent adult obesity. Future research should seek to understand the risk and protective factors associated with overweight/obesity within this understudied population.

## Figures and Tables

**Table 1:** Demographic characteristics of the study sample.

Characteristic	*n*	%	US-Born		Foreign-Born	
			*n*	%	*n*	%
**Sex**						
Male	769	51.3	663	51.8	106	48.2
Female	731	48.7	617	48.2	114	51.8
**Age in 2007**						
5–8 years	1108	73.9	989	77.3	119	54.1
9–11 years	392	26.1	291	22.7	101	45.9
**Insurance Status** ^1^						
Public insurance/self-pay (no change in 2007 vs. 2017)	1241	82.7	1045	82.3	196	89.1
Private insurance (no change in 2007 vs. 2017)	109	7.3	104	8.2	5	2.3
Public insurance/self-pay in 2007 to Private insurance in 2017	102	6.8	83	6.5	19	8.6
Private insurance in 2007 to public insurance/self-pay in 2017	38	2.5	38	3	0	0
**Place of Birth**						
US-Born	1280	85.3	-	-	-	-
Foreign-Born	220	14.7	-	-	-	-

^1^ 10 individuals had missing values for Insurance Status in 2007 and/or 2017.

**Table 2:** Prevalence of overweight/obesity in 2007 and 2017 in the study sample.

Characteristic	2007		2017	
	*n*	%	*n*	%
Entire Sample (*n* = 1500)	449	29.9	279	18.6
**Sex**				
Male (*n* = 769)	295	38.4	187	24.3
Female (*n* = 731)	154	21.1	92	12.6
**Age**				
5–8 years (*n* = 1108)	322	29.1	201	18.1
9–11 years (*n* = 392)	127	32.4	78	19.9
**Place of Birth**				
US-Born (*n* = 1280)	395	30.9	243	19.0
Foreign-Born (*n* = 220)	54	24.5	36	16.4

**Table 3:** McNemar’s test assessing change in proportion of overweight/obese status in the study sample from 2007 to 2017.

Overweight/Obese in 2007	Overweight/Obese in 2017		Total
	**No**	**Yes**	
**No**	977	74	1051
**Yes**	244	205	449
**Total**	1221	279	1500

χ^2^ = 89.814. *p* < 0.001.

**Table 4:** Weight status in 2007 and probability of becoming overweight/obese in 2017.

Characteristic	Weight Status in 2007		
	Underweight/Normal Weight	Overweight/Obese	Adjusted OR ^1^ (95% CI), *p* < 0.001
Entire Sample (*n* = 1500)	7.0%	45.7%	10.30 (7.60–13.96)
**Place of Birth**			
US Born (*n* = 1280)	7.8%	44.1%	8.64 (6.28–11.90)
Foreign Born (*n* = 220)	3.0%	57.4%	40.57 (14.27–115.39)

^1^ Adjusted by sex (i.e., the only variable that was found to be associated with weight status).

## Data Availability

The data presented in this study are not publicly available due to privacy restrictions.
